# The molecular characteristics could supplement the staging system of pT2/T3N0M0 esophageal squamous cell carcinoma: a translational study based on a cohort with over 20 years of follow-up

**DOI:** 10.1186/s12935-024-03286-5

**Published:** 2024-03-30

**Authors:** Wen-Mei Jiang, Jia-Yuan Tian, Yi-Han Guo, Li-Hong Qiu, Xing-Yu Luo, Yang-Yu Huang, Hao Long, Lan-Jun Zhang, Peng Lin, Xin-Xin Xu, Lei-Lei Wu, Guo-Wei Ma

**Affiliations:** 1 State Key Laboratory of Oncology in South China, Collaborative Innovation Center for Cancer Medicine, Sun Yat-sen University Cancer Center, Guangzhou, 510030 P. R. China; 2 Department of Scientific Research, Shaanxi Academy of Social Sciences, Xi’an, 710065 China; 3 Central Hospital of Minhang District, Shanghai, 201100 P. R. China; 4 Zhejiang Cancer Hospital, Hangzhou Institute of Medicine (HIM), Chinese Academy of Sciences, Hangzhou, China; 5 Department of Thoracic Surgery, Zhejiang Cancer Hospital, Hangzhou Institute of Medicine (HIM), Chinese Academy of Sciences, Hangzhou, China

**Keywords:** Esophageal squamous cell carcinoma, Molecular score, Nomogram, Survival

## Abstract

**Objective:**

This study aimed to construct a model based on 23 enrolled molecules to evaluate prognoses of pT2/3N0M0 esophageal squamous cell carcinoma (ESCC) patients with up to 20 years of follow-up.

**Methods:**

The lasso-Cox model was used to identify the candidate molecule. A nomogram was conducted to develop the survival model (molecular score, MS) based on the molecular features. Cox regression and Kaplan-Meier analysis were used in this study. The concordance index (C-index) was measured to compare the predicted ability between different models. The primary endpoint was overall survival (OS).

**Results:**

A total of 226 patients and 23 proteins were enrolled in this study. Patients were classified into high-risk (MS-H) and low-risk (MS-L) groups based on the MS score of 227. The survival curves showed that the MS-L cohort had better 5-year and 10-year survival rates than the MS-H group (5-year OS: 51.0% vs. 8.0%; 10-year OS: 45.0% vs. 5.0%, all *p* < 0.001). Furthermore, multivariable analysis confirmed MS as an independent prognostic factor after eliminating the confounding factors (Hazard ratio 3.220, *p* < 0.001). The pT classification was confirmed to differentiate ESCC patients’ prognosis (Log-rank: *p* = 0.029). However, the combination of pT and MS could classify survival curves evidently (overall *p* < 0.001), which showed that the prognostic prediction efficiency was improved significantly by the combination of the pT and MS than by the classical pT classification (C-index: 0.656 vs. 0.539, *p* < 0.001).

**Conclusions:**

Our study suggested an MS for significant clinical stratification of T2/3N0M0 ESCC patients to screen out subgroups with poor prognoses. Besides, the combination of pT staging and MS could predict survival more accurately for this cohort than the pT staging system alone.

**Supplementary Information:**

The online version contains supplementary material available at 10.1186/s12935-024-03286-5.

## Introduction

Esophageal cancer (EC) is a relatively common malignant tumor of the digestive system in human beings, and according to statistics, there were 572,034 new cases (3.2%) and 508,585 deaths (5.3%) of EC globally in 2018 [1]. Esophageal squamous carcinoma (ESCC), as one of the main pathological types, accounted for up to 90% of EC, with obvious geographic distribution characteristics [2]. Traditional treatment of ESCC includes endoscopic esophageal mucosal dissection, surgical resection, and radiotherapy [3, 4]. With the recent exploration of the pathogenesis, the discovery of various biomarkers, and the application of immunotherapy, the life expectancy of EC patients has improved to a certain extent, but the overall 5-year survival rate remains between 15% and 25% [5]. Tumor staging is crucial for the development of effective treatment strategies and the evaluation of prognosis. The American Joint Committee on Cancer (AJCC) tumor-node-metastasis (TNM) system is a universally adopted language for oncology, and it is widely used as a classical means of prognostic evaluation [6]. However, EC is highly heterogeneous [7–9]. Patients with the same TNM stage may have inconsistent clinical outcomes, and even the 5-year survival rate of patients with cT1N0 stage is worse than that of patients with cT1N+ [10]. Therefore, there is an urgent need to develop a more accurate prognostic model to facilitate the clinical evaluation.

Molecular markers, as useful adjuncts to the TNM staging system, have been continuously explored and tested in recent years [9, 11]. With the widespread clinical use of RNA sequencing and second-generation sequencing, more and more esophageal squamous carcinomas have clear molecular profiles. However, there is still a lack of well-established and relatively well-recognized molecular markers to reveal the prognostic characteristics of patients.

Previous studies showed that the disruption of apoptosis and cell cycle is an important factor in tumor formation [12–15]. The *BCL2* family comprises key regulators of apoptosis, and the high expression of genes in the *BCL2* family could promote the cell proliferation of ESCC [12, 13, 16]. In addition, cell cycle-related proteins, such as Rb, p53, and PCNA, play important roles in regulating the cell proliferation [17, 18]. Therefore, we selected three apoptosis-related (Bcl-2, Bcl2-L-4, and Caspase-3) and five cell cycle-related (Rb, p53, p27^Kip1^, p16^INK4^, and PCNA) antibodies further to explore the prognostic significance of those proteins in ESCC. The WNT signaling pathway (c-Myc, BCL-1, and Catenin beta-1 were selected in this study) and the PI3K-AKT signaling pathway (SPP-1, erbB-2, and EGFR) were selected in this study) can regulate the proliferation and metastasis of esophageal cancer cells, thus implying that proteins on those signaling pathways have the potential to indicate the prognostic characteristics of patients [19, 20]. Previous reports suggested that proteins of pathways in cancer (such as MMP-2, MMP-9, and COX2) and protein family (such as TIMP-2, TIMP-1, and Ki-67) were highly differentially expressed on esophageal cancer and para-cancerous tissues [21–23]. Accordingly, we utilized the proteins associated with the above pathways to explore the prognostic characteristics of the patients.

Most of the studies have been conducted with limited follow-up periods and diverse treatment modalities, thus interfering with the predictive power of molecular models. Therefore, in this study, the expression levels of 23 enrolled molecules were evaluated in pT2/T3N0M0 ESCC patients with up to 20 years of follow-up, in order to more accurately predict the prognosis and stratify the death risk of patient.

## Methods

### Patients selection

**Fig. 1:**
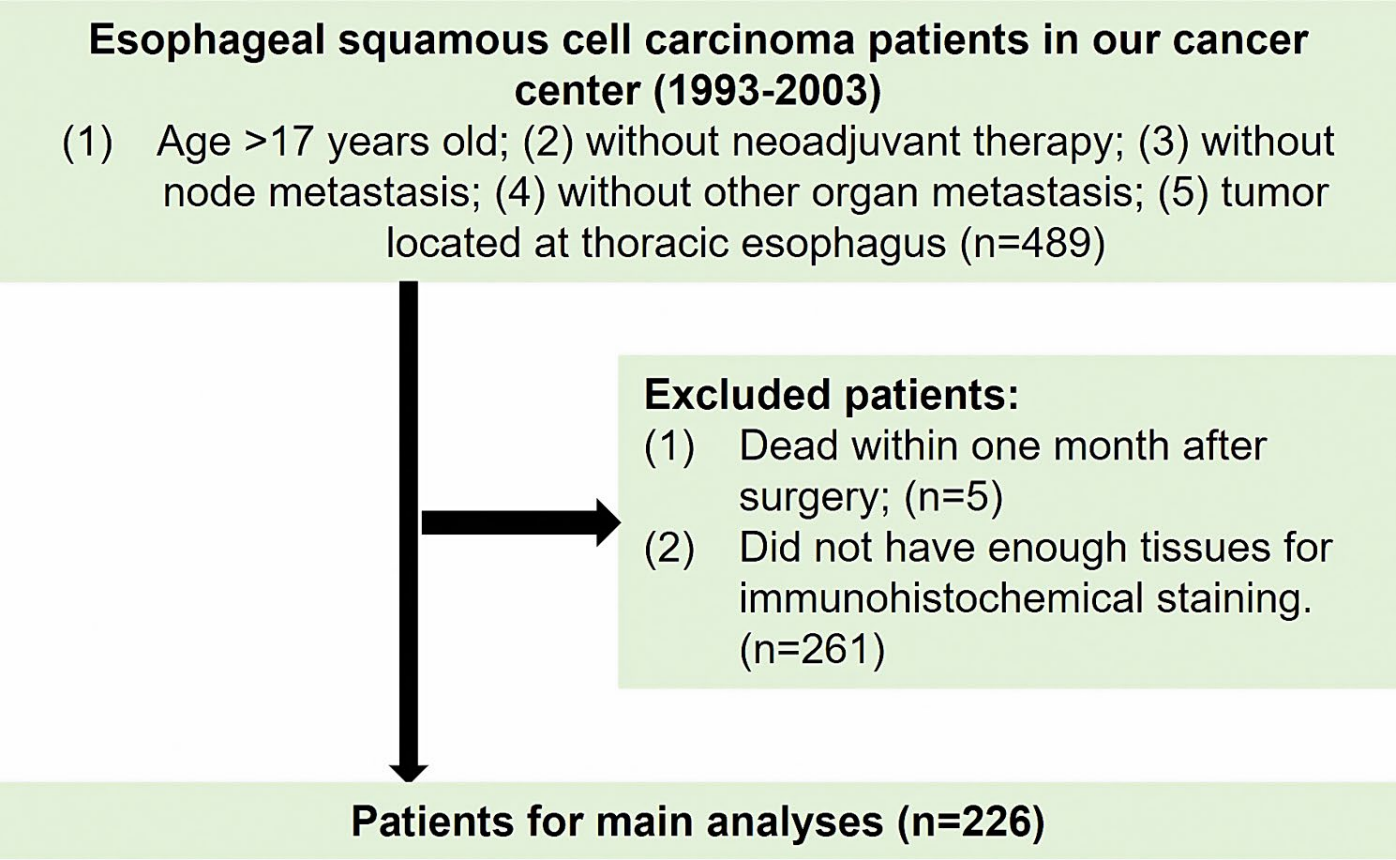
The flowchart of patient selection

The study was conducted in accordance with the Declaration of Helsinki (as revised in 2013). Our study was composed of patients who underwent surgery for primary ESCC at Sun Yat-sen University Cancer Center between 1993 and 2003. The selection criteria of patients are presented in Fig. 1. A total of 226 patients were enrolled, and the TNM stage was identified according to the eighth edition TNM classification system. Patient’s clinical and pathological data included age at surgery, sex, tumor location, surgery, pathological T (pT) stage, and tumor grade.

### Follow-up and outcomes

All patients were followed up after surgery, and the relevant information was obtained through telephone calls or medical records. The latest follow-up was updated on 24th May 2023. Overall survival (OS) was calculated from the date of surgery to the date of death or the last day of follow-up.

### Immunohistochemical staining

Tumor and non-tumor paraffin-embedded tissues of all 226 patients were performed according to an Envision system of manufacturer’s instructions (Glostrup, Dako, Denmark). We selected proteins from apoptosis pathway (Bcl-2, Bcl2-L-4, and Caspase-3), WNT signaling pathway (c-Myc, BCL-1, and Catenin beta-1), pathways in cancer (MMP-2, MMP-9, PTEN, Cadherin-1, and COX2), cell cycle (p16^INK4^, Rb, p53, PCNA, and p27^Kip1^), PI3K-AKT signaling pathway (SPP-1, EGFR, and erbB-2), protein families (HSP70, Ki-67, TIMP-1, TIMP-2, Galectin-3, and p63), and others (ID-1 and CD44v6) to use in this study. The detailed information about 27 primary antibodies is presented in Supplementary Table 1. Ventana OmniMap anti-rabbit and anti-rat antibodies were used as secondary antibodies. Staining intensity and extent were scored as previously reported [9]. The intensity of immunostaining was calculated as point 0 for no staining, 1 for very weak staining, 2 for moderate staining, and 3 for intense staining. The extent of immunoreactive tumor cell scoring was evaluated as follows: 0 for 0% positive cells, 1 for 1–10% positive cells, 2 for 11–25% positive cells, 3 for 26–40% positive cells, and 4 for over 41% positive cells. The final quantitation for each staining was obtained by multiplying the two points mentioned above. The results of immunohistochemical staining were interpreted independently by two pathologists under double-blind conditions who didn’t know any clinical or other pathological information about patients. When the results were inconsistent, they would perform a joint discussion to decide the final results.

### Statistical analysis

The cut-off values of protein expression and molecular scores were calculated by R version 4.1.1 (https://www.r-project.org/). Besides, the Lasso-Cox, Kaplan-Meier, and nomogram analyses were conducted by R version 4.1.1. Other analyses were performed using the software SPSS 25.0 (IBM SPSS, Inc., Armonk, NY). The proportions of categorical outcomes were assessed by Pearson’s Chi-square test or Fisher’s exact test. Univariable and multivariable Cox proportional hazard models were adopted to calculate the hazard ratio (HR) and 95% confidence interval (CI). Predictors (*p* < 0.2) in univariable analysis and the known affecting-prognosis factors were brought into a multivariable analysis. The statistical significance was considered as a *p* < 0.05 on two sides. The concordance index (C-index) was measured to compare the predicted ability between different models. OS was analyzed by using the Kaplan-Meier method, and the differences were compared by log-rank tests. The primary endpoint was overall survival.

## Results

### Patient characteristics

In this study, men outnumbered women, constituting 72.5% of the patients. A total of 175 (77.4%) patients were aged 65 and below, whereas 51 (22.6%) were over 65. The majority of patients were diagnosed with grade I or II ESCC, comprising more than 75% of the whole cohort. In terms of the pT classification, most patients were diagnosed with T3 ESCC. The number of patients with low molecular score (MS-L) (*n* = 105) was smaller than that of patients with high molecular score (MS-H) (*n* = 121). The baseline characteristics of the enrolled group are shown in Table 1.

**Table 1:** The baseline information of esophageal squamous cell carcinoma

	Molecular score		${\varvec{\upchi }}^{2}$	p-value
	Low (*n* = 105)	High (*n* = 121)		
**Sex**			0.003	0.954
Male	76(72.4%)	88(72.7%)		
Female	29(27.6%)	33(27.3%)		
**Age**			2.244	0.134
<=65	86(81.9%)	89(73.6%)		
> 65	19(18.1%)	32(26.4%)		
**Tumor location**			0.672	0.715
Upper	10(9.5%)	15(12.4%)		
Middle	70(66.7%)	81(66.9%)		
Lower	25(23.8%)	25(20.7%)		
**Surgical approach**			6.001	0.423*
Sweet	76(72.4%)	93(76.8%)		
Two incisions in the left chest	4(3.8%)	1(0.8%)		
Ivor-Lewis	3(2.9%)	5(4.1%)		
McKeown	22(20.9%)	22(18.3%)		
**Examined lymph nodes**			0.009	0.924
≤ 6	54(51.4%)	63(52.1%)		
> 6	51(48.6%)	58(47.9%)		
**Tumor grade**			8.489	0.014
Grade I	33(31.4%)	24(19.8%)		
Grade II	55(52.4%)	59(48.8%)		
Grade III	17(16.2%)	38(31.4%)		
**Tumor length (cm)**			0.017	0.897
≤ 5	72(68.6%)	82(67.8%)		
> 5	33(31.4%)	39(32.2%)		
**pT**			0.093	0.760
T2	42(40.0%)	46(38.0%)		
T3	63(60.0%)	75(62.0%)		

*This comparison was based on the Fisher’s exact test. Other comparisons were based on the Chi-square test

### Lasso-Cox regression analyses of proteins

**Fig. 2:**
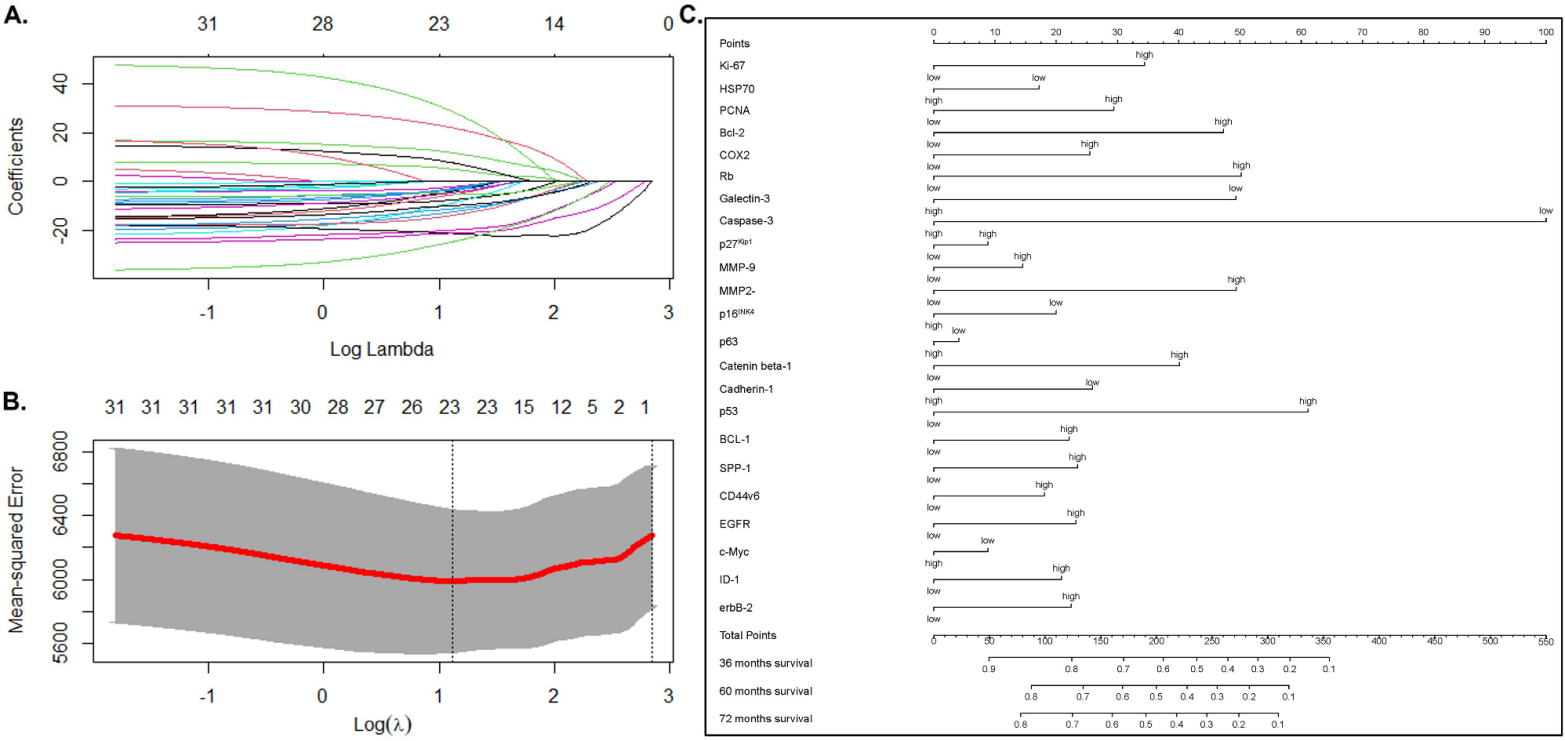
The procession of Lasso-Cox model (**A** and **B**). The nomogram based on selected proteins from the Lasso-Cox model (**C**)

The cutoff values of proteins were calculated by R software and recorded in Supplementary Table 2, by which the patients were divided into low- and high-expression groups. LASSO regression analysis was performed on the whole 27 proteins with prognostic value, and the risk coefficient of each protein was evaluated. Finally, twenty-three prognostic proteins were retained according to the minimum partial likelihood of deviance (Fig. 2A, B).

### The development of a nomogram

**Fig. 3:**
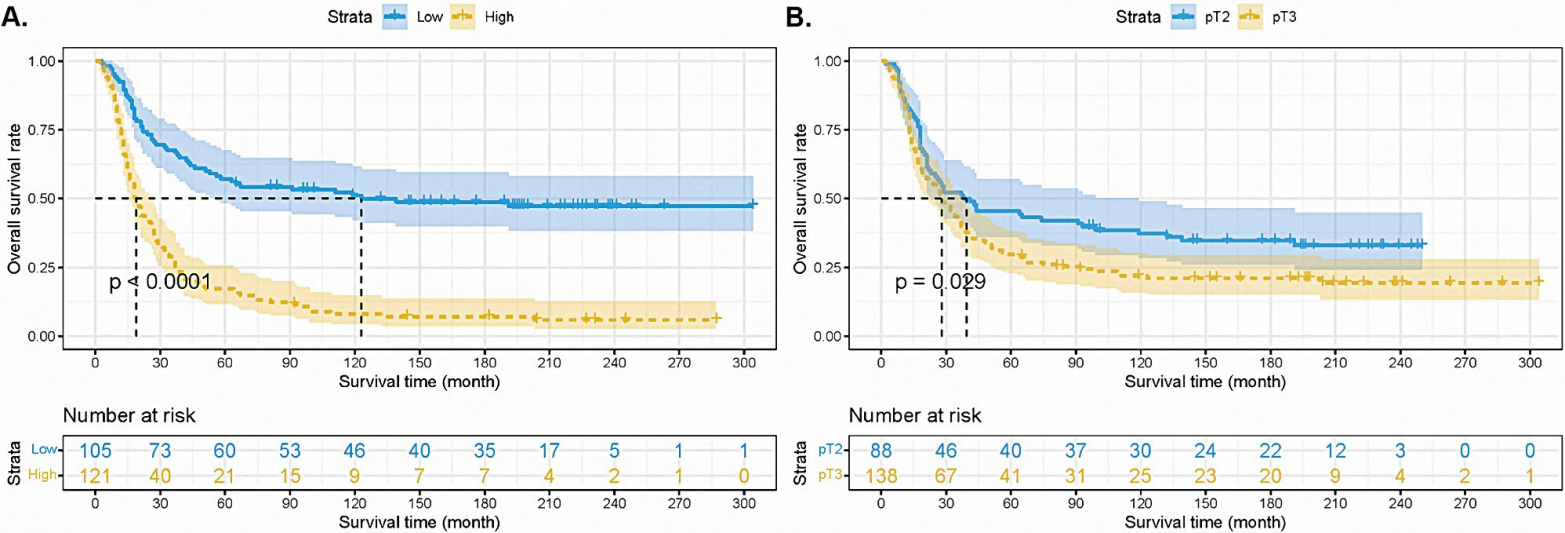
The survival curves based on the molecular score (**A**) and the pT classification (**B**)

We used the 23 proteins to construct a nomogram (Fig. 2C). The point of every protein is listed in Table 2. Then, we set a MS based on the points of 23 proteins. According to the analysis of R software, the cutoff value of MS was identified as 227. The survival curves were drawn by Kaplan-Meier analysis and showed that the cohort with MS-L had better 5-year and 10-year survival rates than the MS-H group (5-year OS: 51.0% vs. 8.0%; 10-year OS: 45.0% vs. 5.0%, all *p* < 0.001, Fig. 3A).

**Table 2:** The point of every protein screened by Lasso-Cox analyses

Protein	Points	Protein	Points	Protein	Points
Ki-67		p27^Kip1^		BCL-1	
low	0	low	0	low	0
high	34	high	9	high	22
HSP70		MMP-9		SPP-1	
low	17	low	0	low	0
high	0	high	15	high	23
PCNA		MMP-2		CD44v6	
low	0	low	0	low	0
high	29	high	49	high	18
Bcl-2		p16^INK4^		EGFR	
low	0	low	20	low	0
high	47	high	0	high	23
COX2		p63		c-Myc	
low	0	low	4	low	9
high	25	high	0	high	0
Rb		Catenin beta-1		ID-1	
low	0	low	0	low	0
high	50	high	40	high	21
Galectin-3		Cadherin-1		erbB-2	
low	49	low	26	low	0
high	0	high	0	high	22
Caspase-3		p53			
low	100	low	0		
high	0	high	61		

### Univariable and multivariable analyses

The outcomes of univariable and multivariable analyses are presented in Table 3. In order to discriminate the prognostic factors, a total of 6 variables were included in the univariable Cox regression analysis. MS (*p* < 0.001) and pT (*p* = 0.036) were considered to aggravate the survival. Furthermore, multivariable analysis confirmed MS as an independent prognostic factor after eliminating the confounding factors (HR = 3.220, *p* < 0.001).

**Table 3:** Univariable and multivariable analyses for the cohort

Variables	Univariable Analysis				Multivariable Analysis			
	HR	95% CI	β	p-value	HR	95% CI	β	p-value
**Sex**								
Male	1	Reference			1	Reference		
Female	0.754	0.530–1.074	−0.282	0.118	0.734	0.505–1.068	−0.309	0.106
**Age**								
≤ 65	1	Reference			1	Reference		
> 65	1.344	0.947–1.908	0.296	0.098	1.245	0.851–1.820	0.224	0.259
**Surgical approach**								
Sweet	1	Reference						
Two incisions in the left chest	0.880	0.325–2.384	−0.128	0.802				
Ivor-Lewis	1.394	0.650–2.990	0.332	0.393				
McKeown	1.203	0.819–1.767	0.184	0.347				
**Examined lymph nodes**								
≤ 6	1	Reference						
> 6	0.936	0.691–1.269	−0.066	0.671				
**Tumor location**								
Upper	1	Reference			1	Reference		
Middle	0.697	0.433–1.124	−0.360	0.139	0.640	0.393–1.042	−0.446	0.073
Lower	0.688	0.400–1.184	−0.373	0.177	0.594	0.342–1.032	−0.521	0.064
**Tumor grade**								
Grade I	1	Reference		0.081	1	Reference		
Grade II	1.024	0.698–1.500	0.023	0.904	0.923	0.627–1.359	−0.098	0.686
Grade III	1.495	0.977–2.289	0.402	0.064	1.219	0.785–1.892	0.165	0.377
**Tumor length (cm)**								
≤ 5	1	Reference						
> 5	1.014	0.733–1.402	0.014	0.934				
**pT**								
T2	1	Reference			1	Reference		
T3	1.421	1.032–1.957	0.352	0.031	1.328	0.960–1.837	0.283	0.087
**Molecular Score**								
Low	1	Reference			1	Reference		
High	3.393	2.432–4.734	1.222	< 0.001	3.263	2.321–4.586	1.183	< 0.001

### Combination of pT and MS

**Fig. 4:**
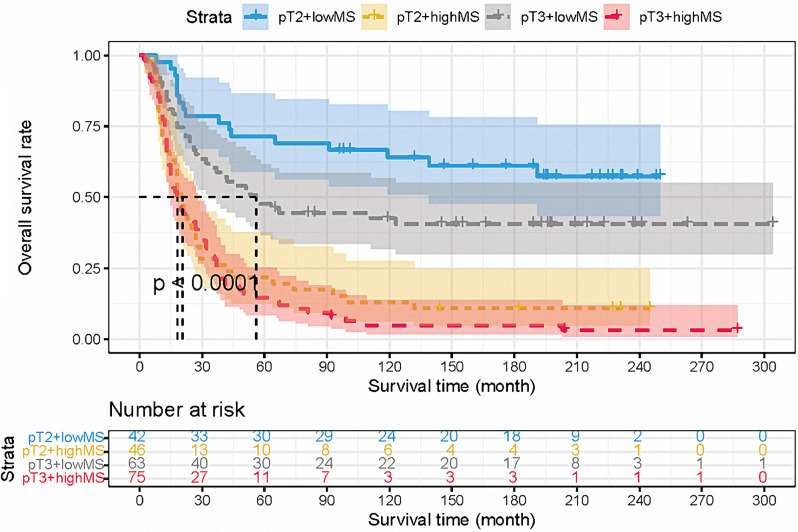
The survival curves based on the combination of molecular score and pT classification

The pT classification, as a classical indicator, was confirmed to be able to differentiate the prognosis of ESCC patients by Kaplan-Meier analysis (Log-rank: *p* = 0.029, Fig. 3B). However, the combination of pT and MS could classify survival curves evidently (overall *p* < 0.001, Fig. 4). Besides, Table 4 showed the univariable analysis based on the combination of the pT and MS. We calculated the C-index to compare the predictive ability between pT alone and the combination of the pT and MS, which showed that the prognostic prediction efficiency was improved significantly by the combination of the pT and MS (C-index = 0.656, SE = 0.02) than by the classical pT classification (C-index = 0.539, SE = 0.02, *p* < 0.001).

**Table 4:** Univariable analysis for the combination of pT and MS

Variable	Univariable Analysis		p-value
	HR	95% CI	
pT3 + low MS vs. pT2 + low MS	1.809	1.017–3.216	0.044
pT2 + high MS vs. pT3 + low MS	2.340	1.492–3.670	< 0.001
pT3 + high MS vs. pT2 + high MS	1.182	0.803–1.739	0.397

## Discussion

In the present study, we collected the data of ESCC patients, including clinical, pathological, and protein expression information. We performed the Lasso-Cox regression, Cox regression, and nomogram to explore the predictive effect of protein expression in ESCC patients. Then, we constructed a molecular score based on the protein impacting prognosis. After adjusting for other confounders, the molecular score was identified as an independent factor, which could classify survival curves evidently. We combined the MS and TNM staging system to predict the survival of ESCC patients. Overall, the predictive ability was better in the combination of the MS and pT staging system than in the traditional pT staging system alone. Therefore, we suggest that the molecular characteristics could improve the prognostic predictive of ESCC patients. Besides, those results inform the inclusion of molecular features in the future TNM staging system of ESCC.

It’s important to evaluate the molecular features of ESCC patients. Previous studies from basic experiments found that the expression of *MMP2* could affect the migration, invasion, and metastasis of ESCC cells [24, 25]. A study from *Li et al.* reported that the expression of MMP2 in ESCC was significantly associated with tumor invasion depth and lymph node metastasis [26]. Tumor invasion and lymph node metastasis imply high levels of T and N classification and a poor prognosis. However, there was no survival analysis in their research. Besides, the abovementioned study had a small sample size (*n* = 58) but included complex TNM staging that encompassed stages I-III diseases, making it difficult to state that the same stage carrying different genetic features would have different prognostic characteristics. In the present study, we provided the prognostic data over 20 years and confirmed that T2/3N0M0 ESCC patients with the higher expression of MMP2 had poorer survival. Recently, COX2 has become the hotspot in anti-cancer research, and it has been demonstrated that COX2 could play an important role in tumor promotion and the sensitivity of immunotherapy [27–29]. High expression of COX2 can promote the proliferation, angiogenesis, invasiveness, metastasis, and also the inhibition of apoptosis and immuno-surveillance [27, 30]. A brief report found that a combination of acetaminophen (COX1/2 inhibitor) and anti-PD-1 treatment significantly reduced the treatment efficacy compared with anti-PD-1 treatment alone in the Lewis lung carcinoma mouse model [28]. In our study, the high expression of COX2 indicated worse outcomes for ESCC patients. Therefore, these results suggest that molecular signatures not only provide an indication of a patient’s prognosis but also serve as an indicator of therapeutic efficacy or as a potential target for therapy.

Adding molecular characterization to clinical data really improves the stratification of patient prognosis. In our previous study, the improvement in the accuracy of prognostic modeling with the addition of the assessment of PD-L1 expression levels was significant (C-index increased by 0.204) [9]. Another study by us showed that the expression of FOXD1 could serve as an independent prognosticator for head and neck squamous cell carcinoma, which indicated that adding the expression of FOXD1 into the prognostic assessment tool, such as TNM system, could improve the prognostic prediction [31]. A report by *Liu et al.* found that *CAV1* promoted glioma progression, and its high expression was related to the poor prognosis [32]. Similarly, the expression of *CAV1* also indicated the prognosis independently. A study by *Tang et al.* confirmed that the high expression of RRM2 enhanced esophageal cancer’s radiotherapy resistance and shortened those patients’ survival [33]. Those studies mentioned above demonstrate the value of molecular profiling in the prognostic assessment of patients with malignancies. The nomogram, as an individualized scoring tool, is useful in clinical practice. The development and use of nomogram has improved cancer patients’ prognostic assessment [34–37]. In this study, we used both of these tools: molecular characterization and nomogram. Importantly, our follow-up is over 20 years, giving our model better prognostic prediction stability.

This study still has some drawbacks. First, the sample size was relatively small, although all patients belonged to T2/3N0M0 diseases. Further large multicentric studies are required to research and verify our results. Second, Although the follow-up period was long, the recurrence of the patients was difficult to document, and therefore, it is not possible to give information on the role of molecular features on disease recurrence in this study. Third, we chose 27 proteins, but we did not explore the interrelationships between these proteins. Fourth, considering the inherent shortcomings of retrospective studies, there is still a need for prospective studies to further explore the significance that molecular characterization brings to patient prognostic assessment and therapeutic decision-making.

## Conclusions

Our study suggested an MS for significant clinical stratification of T2/3N0M0 ESCC patients to screen out subgroups with poor prognoses. Besides, the combination of TNM staging and MS could predict survival more accurately for this cohort than the pT staging system alone.

### Electronic supplementary material

Below is the link to the electronic supplementary material.

**Supplementary Material 1: Supplementary Table 1.** The detailed information about 27 primary antibodies

**Supplementary Material 2: Supplementary Table 2.** The cutoff values of genes in this study.

## Data Availability

Any researchers interested in this study could contact corresponding author for requiring data.
