# Supplementary material for: The molecular characteristics could supplement the staging system of pT2/T3N0M0 esophageal squamous cell carcinoma: a translational study based on a cohort with over 20 years of follow-up
Source: Cancer Cell Int. 2024 Mar 30;24:119. doi: 10.1186/s12935-024-03286-5 (PMC10981364; doi:10.1186/s12935-024-03286-5)
Supplement: Supplementary file 1 — Supplementary Material 1: Supplementary Table 1. The detailed information about 27 primary antibodies [file 12935_2024_3286_MOESM1_ESM.docx]

**Supplementary Table 1. The detailed information about 27 primary antibodies.**

| **Primary**  **antibody** | **Clone** | **Dilution** | **Source** |
| --- | --- | --- | --- |
| **Apoptosis pathway** | | | |
| Bcl-2 | rab monoclone | 1: 100 | RMA-0660, Maixin Ltd., Fuzhou, China |
| Bcl2-L-4 | rat monoclone | 1: 100 | MAB-0243, Maixin Ltd., Fuzhou, China |
| Caspase-3 | rab polyclone | 1: 100 | AF6311, Affinity Biosciences Ltd., Liyang, China |
| **WNT signaling pathway** | | | |
| c-Myc | rab polyclone | 1：30 | RMA-0803, Maixin Ltd., Fuzhou, China |
| BCL-1 | rab monoclone | 1: 100 | RMA-0541, Maixin Ltd., Fuzhou, China |
| Catenin beta-1 | rat monoclone | 1: 100 | MAB-0754, Maixin Ltd., Fuzhou, China |
| **Pathways in cancer** | | | |
| MMP-2 | rab polyclone | 1: 25 | AF5330, Affinity Biosciences Ltd., Liyang, China |
| MMP-9 | rab polyclone | 1: 30 | AF5228, Affinity Biosciences Ltd., Liyang, China |
| PTEN | rab monoclone | 1: 100 | RMA-1074, Maixin Ltd., Fuzhou, China |
| Cadherin-1 | rat monoclone | 1: 100 | MAB-0738, Maixin Ltd., Fuzhou, China |
| COX2 | rab monoclone | 1: 100 | RMA-0549, Maixin Ltd., Fuzhou, China |
| **Cell cycle** | | | |
| p16^INK4^ | rat monoclone | 1: 100 | MAB-0673, Maixin Ltd., Fuzhou, China |
| Rb | rat monoclone | 1: 100 | MAB-0186, Maixin Ltd., Fuzhou, China |
| p53 | rat monoclone | 1: 150 | MAB-0647, Maixin Ltd., Fuzhou, China |
| PCNA | rat monoclone | 1: 100 | MAB-0145, Maixin Ltd., Fuzhou, China |
| p27^Kip1^ | rat monoclone | 1: 100 | MAB-0793, Maixin Ltd., Fuzhou, China |
| **PI3K-AKT signaling pathway** | | | |
| SPP-1 | rab polyclone | 1: 100 | YBA899Bo01, Yubo Biotechnology Co. Ltd., Shanghai, China |
| EGFR | rat monoclone | 1: 100 | RMA-0804, Maixin Ltd., Fuzhou, China |
| erbB-2 | rab monoclone | 1: 100 | Kit-0043, Maixin Ltd., Fuzhou, China |
| **Protein families** | | | |
| HSP70 | rab polyclone | 1: 50 | AF5466, Affinity Biosciences Ltd., Liyang, China |
| Ki-67 | rat monoclone | 1: 100 | MAB-0672, Maixin Ltd., Fuzhou, China |
| TIMP-1 | rab polyclone | 1: 40 | AF7007, Affinity Biosciences Ltd., Liyang, China |
| TIMP-2 | rab polyclone | 1: 50 | AF7008, Affinity Biosciences Ltd., Liyang, China |
| Galectin-3 | rat monoclone | 1: 100 | MAB-0835, Maixin Ltd., Fuzhou, China |
| p63 | rat monoclone | 1：200 | MAB-0694, Maixin Ltd., Fuzhou, China |
| **Others** | | | |
| ID-1 | rab polyclone | 1: 100 | DF2932, Affinity Biosciences Ltd., Liyang, China |
| CD44v6 | rat monoclone | 1: 100 | MAB-0038, Maixin Ltd., Fuzhou, China |

Rab: rabbit.
