# Supplementary material for: The molecular characteristics could supplement the staging system of pT2/T3N0M0 esophageal squamous cell carcinoma: a translational study based on a cohort with over 20 years of follow-up
Source: Cancer Cell Int. 2024 Mar 30;24:119. doi: 10.1186/s12935-024-03286-5 (PMC10981364; doi:10.1186/s12935-024-03286-5)
Supplement: Supplementary file 2 — Supplementary Material 2: Supplementary Table 2. The cutoff values of genes in this study. [file 12935_2024_3286_MOESM2_ESM.docx]

**Supplementary Table 2.** The cutoff values of proteins in this study.

| **Protein** | **Cutoff value** | **Protein** | **Cutoff value** |
| --- | --- | --- | --- |
| Bcl2-L-4 | 0.667 | MMP-2 | 5.000 |
| Bcl-2 | 7.333 | MMP-9 | 5.333 |
| COX2 | 10.000 | SSP-1 | 5.333 |
| Caspase-3 | 4.667 | PCNA | 8.667 |
| BCL-1 | 3.000 | PTEN | 3.667 |
| CD44v6 | 14.000 | p16^INK4^ | 4.667 |
| c-Myc | 2.000 | p63 | 12.000 |
| Cadherin-1 | 3.667 | p27^Kip1^ | 1.000 |
| EGFR | 6.000 | p53 | 13.000 |
| Galectin-3 | 0.000 | Rb | 12.000 |
| erbB-2 | 4.333 | TIMP-1 | 4.333 |
| HSP70 | 11.667 | TIMP-2 | 11.000 |
| ID-1 | 0.333 | Catenin beta-1 | 0.333 |
| Ki-67 | 10.000 | Molecular score | 227.000 |
